# Material characterization and *Streptococcus oralis* adhesion on Polyetheretherketone (PEEK) and titanium surfaces used in implantology

**DOI:** 10.1007/s10856-020-06408-3

**Published:** 2020-09-28

**Authors:** Simonetta D’Ercole, Luigina Cellini, Serena Pilato, Silvia Di Lodovico, Giovanna Iezzi, Adriano Piattelli, Morena Petrini

**Affiliations:** 1 Department of Medical, Oral and Biotechnological Sciences, University of Chieti, Via dei Vestini 31, 66100 Chieti, Italy; 2 Department of Pharmacy, University of Chieti, Via dei Vestini 31, 66100 Chieti, Italy; 3 Fondazione Villa Serena per la Ricerca, Città S. Angelo, Via Petruzzi 42, 65013 Chieti, Italy

## Abstract

The aim of this study was to evaluate the interaction between *Streptococcus oralis* and Polyetheretherketone (PEEK), a novel material recently introduced in implantology. The topographical characterization and the *Streptococcus oralis* adhesion on this material were compared with other titanium surfaces, currently used for the production of dental implants: machined and double etched (DAE). The superficial micro-roughness of the PEEK discs was analyzed by scanning electron microscopy (SEM) and, the Energy Dispersive Spectrometer (EDS) analyzed their chemical composition. Atomic Force Microscopy (AFM) was used to characterize the micro-topography and the sessile method to evaluate the wettability of the samples. Microbiological analysis measured the colony forming units (CFUs), the biomass (OD_570_ detection) and the cell viability after 24 and 48 h after *Streptococcus oralis* cultivation on the different discs, that were previously incubated with saliva. Results showed that PEEK was characterized by a micro-roughness that was similar to machined titanium but at nano-level the nano-roughness was significantly higher in respect to the other samples. The EDS showed that PEEK superficial composition was characterized mainly by Carbonium and Oxygen. The hydrophilicity and wetting properties of PEEK were similar to machined titanium; on the contrary, double etched discs (DAE) samples were characterized by significantly higher levels (*p* < 0.05). PEEK was characterized by significant lower CFUs, biomass and viable cells in respect to the titanium surfaces. No differences were found between machined and DAE. The anti-adhesive and antibacterial properties showed by PEEK at 24 and 48 h against a pioneer such as *S. oralis*, could have an important role in the prevention of all pathologies connected with biofilm formation, like peri-implantitis in dentistry or prosthetic failures in orthopedics.

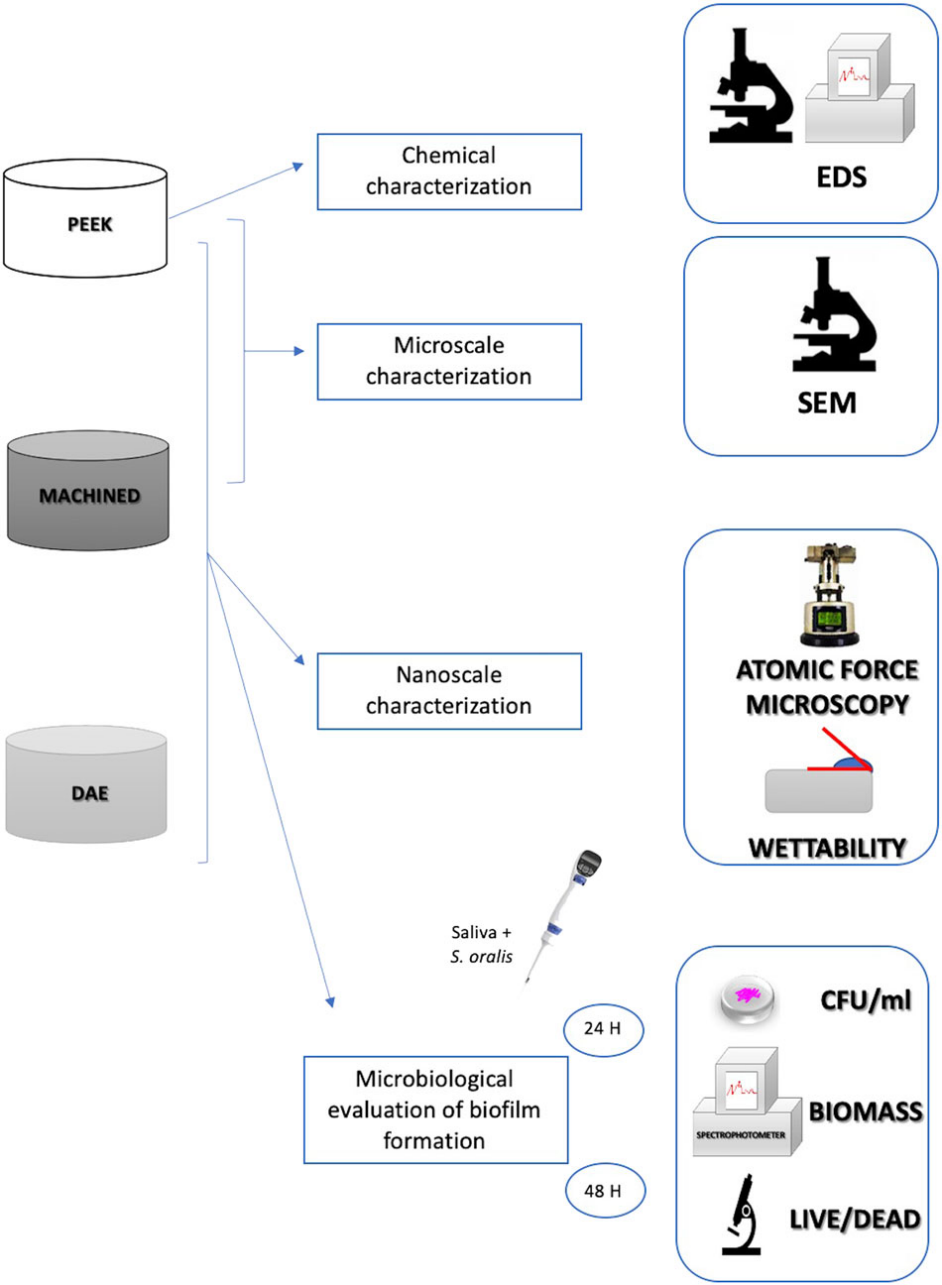

## Introduction

Research in implantology has mainly focused on novel materials and technologies that could prevent or treat diseases in order to minimize the loss of teeth and their support, or to find biomimetic materials that could substitute the natural elements for function and esthetics [1–3]. Among these, Polyetheretherketone, PEEK, is a thermoplastic polyaromatic semi crystalline polymer with a temperature fusion of about 340 °C [4]. The clinical use of this polymer in trauma, orthopedic surgery and spinal implants dates back to the 1990s and, in vitro and in vivo, studies on dental research have focused on the comparison between titanium and PEEK effects on bone formation, in order to verify its use in implantology [5]. PEEK has a potential use in this field, thanks to its tensile and Young’s elastic modulus (3–4 GPa) that are similar to human bone and dentine, contrarily to those of titanium that are higher [6]. Moreover, another advantage of PEEK is the possibility to modify its mechanical properties, by filling or mixing it with other elements, like carbonium fibers, increasing the elastic modulus to 18 GPa, that is similar to the 15 GPa cortical bone [4, 7]. Other advantages of PEEK are biocompatibility, chemical and physical stability, esthetics, because its color is similar to the tooth and the possibility to be manufactured by means of 3D printers [8–10]. The use of PEEK in implantology also permits to overlap the problem of titanium hyper sensibility, allergy and other problems connected with ions release [11]. Conventional PEEK is characterized by hydrophobicity and smooth surface due to injection molding or machining processes and, as for titanium, smooth surfaces exhibit less osteoinductivity when compared to topographically more complex ones [12]. However, it has been shown that the superficial characterization of dental implants with macro and micro-porous structures is able to influence both the apposition of bone during neo-osteogenesis and the removal torque, while the macrostructure has more influence on the angiogenesis [13–15]. In previous studies, we have shown that the macroscopic structure of the fixture and the type of implant connection influences the microbiota around dental implants [16, 17]. However, it is also fundamental to consider the great importance of the micro-topography of the implants in the increase of the bone implant contact and the removal torque that decrease early failure rates and permits the reduction of the loading protocols or the immediate prosthetic rehabilitation [15]. Considering the inert nature of PEEK and the poor osteogenic potential, the enrichment with bioactive substance like hydroxyapatite (HA) and tri-calcium phosphate (TCP) or the development of other fabrication modalities of PEEK samples that could increase the porousness of the material, have been studied [5, 12]. A recent review published by Panayotov et al., summarized possible PEEK modifications that permit to increase its biological properties [18]. The changes could be structural (3d configuration), could interest its chemical composition, like the addition of fillers, could interest the whole material as well as only the superficial layers [18]. A porous PEEK, produced by extruding medical-grade PEEK through the lattice spacing of sodium chloride crystals, was associated to increase cell differentiation in osteoblasts, osteocalcin production and mineralization, in respect to smooth PEEK and machined titanium [12].

The good interaction between PEEK polymers and soft tissues has also permitted to use this material for the production of prosthetic components, like healing screws and abutments [19]. However, in order to evaluate a possible use in implantology, it is important to verify the effects of PEEK on bacterial adhesion and biofilm.

During biofilm formation in the oral cavity, initial colonizers utilize the acquired pellicle generated by the saliva and express surface receptors which moderate their adherence to it. Early colonizers of dental biofilm include various streptococci, such as *Streptococcus oralis*, which can adhere directly to the soft tissues of tongue, cheeks, gingiva and palate and on hard surfaces of teeth and bone and bind to other species in the initial biofilm altering their surroundings and creating new niches for other potential pathogens [20].

The role of biofilm is central on peri-implantitis etiology: it is recognized as a complex infection of peri-implant tissues that are colonized by uncultivable asaccharolytic, anaerobic Gram-positive and Gram-negative rods, which are not frequently identified in teeth with periodontitis or healthy implants [21].

The marginal bone resorption and tissues destruction of peri-implantitis are the consequence, in part, of a direct bacterial action, but it is mainly caused by an excessive host response, due to the dysbiosis [22].

The aim of this work is to verify if PEEK interaction with *Streptococcus oralis* could be more performant on bacterial adhesion inhibition in respect to two titanium surfaces used in implantology: machined and double acid etched (DAE). The secondary outcome is the material characterization of the three types of samples, in order to increase the knowledge of the parameters that could affect the bacterial colonization. A correlation between the superficial nano- and micro-structure of the three discs and a microbiological analysis was performed.

## Materials and methods

### Specimen preparation

A total of 144 discs (Kristal-PHI, Trezzano sul Naviglio, Milano, Italy), 48 for each studied material, were used in this in vitro study.

All discs were produced through turning, starting from the milling of a bar characterized by the same diameter of the final discs. Then, they were ultrasound washed with detergent and dried 1 h in the oven at 60 °C.

The experimental specimens have a diameter of 5 mm and a thickness of 2 mm, with three different finished surfaces, corresponding to three groups:

- Titanium (Ti) IV grade (ASTM F67) Machined.
- Titanium IV grade (ASTM F67) double acid etched, DAE: machined discs were subjected to a double acid treatment; the first with a solution containing fluoridric acid and the second with nitric acid.
- PEEK (Tekapeek classic white, Ensinger, Italy).

Before the experiments, all samples were placed in 75% ethanol for 60 min and dried in a sterilized clean bench; both sides of the sample were irradiated with ultraviolet light for 30 min.

The sterile specimens were placed in 96-well polystyrene microtiter plates and prior to each experiment, inoculated for 2 h in saliva at 37 °C in shaking incubator with slight agitation, to allow protein pellicle formation.

### Scanning electron microscope observation (SEM)

A Phenom ProX scanning electron microscope (Phenom-World B.V., The Netherlands) with the Element Identification (EID) software package was used to characterize PEEK samples at microscale and to perform the Energy Dispersive Spectrometer (EDS). Before starting with SEM observation, a Desk Sputter Coater (Phenom-World B.V., The Netherlands) has been used to sputter the PEEK samples with gold (150 A).

### Atomic Force Microscopy (AFM)

The surface nano-roughness (Ra) of the three different surfaces were analyzed by Atomic Force Microscopy (AFM).

The ScanAsyst technique was used for the atomic force microscopy (Bruker) observations with scan size of 10 µm*10 µm and RTESPA-300 probe.

The software Nanoscope was used to analyze images and 3D reconstruction. The roughness average (Ra), that is the arithmetic mean of the absolute values of the height of the surface profile, was considered for the statistical analysis.

Five samples of each group were observed and the mean values (± standard deviation) were considered for the statistical analysis.

### Measurement of wetting properties

The sessile drop method was used to measure the wetting properties of the groups, as previously described [23]. A Nikon D90 DSLR Camera (Nikon Corporation, Tokyo, Japan) with 18–105 mm Lens was used to photograph the samples (Fig. 6). The water contact angle and the wetted area were measured by using Image J 1.52q for Mac OS X (USA). Five samples of each group were observed.

### Microbial strain

A clinical strain of *Streptococcus oralis* CH 05, isolated by saliva sample from healthy individual and collected at the Department of Biomedical Sciences, was used for this study [24]. The strain, stored at −80 °C, was reconstituted in Brain Heart Infusion broth (BHI, Oxoid, Milan, Italy) overnight at 37 °C under anaerobic condition. Overnight cultures were diluted 1:10 in the same medium and refreshed for 2 h at 37 °C in a shaking thermostatic water bath (160 rpm).

Then, the broth culture was standardized to obtain a suspension containing 9 × 10^6^ CFU/mL (optical density/OD_600_ = 0.12) and used for experiments. OD_600_ readings were performed using a spectrophotometer (Eppendorf, Milan, Italy).

### Saliva collection

The human saliva was collected from 4 healthy volunteers (two females and two males), who hadn’t drunken or eaten in at least 2 h. Exclusion criteria included oral diseases (caries or periodontitis), dental care in progress, antibiotics consumption for 3 months prior to the beginning of the study.

Saliva samples were mixed, clarified by centrifugation at 16,000 × *g* for 1 h at 4 °C, and sterilized through low protein binding filters (pore sizes of 0.8 μm, 0.45 µm and 0.2 μm).

The sterility of the saliva was verified by incubation in the Tryptic Soy Agar (TSA, Becton Dickinson, USA) plates [25]. Saliva was considered to be sterile if no growth could be detected in both aerobic and anaerobic atmosphere for 24–48 h at 37 °C [25]. Sterile saliva was stored at −20 °C and processed within 2 days.

### Biofilm development

Biofilm formation was developed as follows: 200 µL of standardized *S. oralis* suspension was added on the protein-coated discs inside 96-well polystyrene microtiter plates and incubated statically at 37 °C for 24 and 48 h under anaerobic condition. Negative controls, consisting of non-inoculated wells containing the tested discs, were also prepared. Subsequently, microbial suspensions were carefully removed and the samples were rinsed 3 times with PBS to remove the planktonic bacteria.

The effect of different implant surfaces on the biofilm formation were determined by (i) the colony-forming units (CFU) count for the quantification of cultivable cells, (ii) the biofilm biomass evaluation by Hucker’s crystal violet staining method, and (iii) the cells viability by Live/Dead analysis.

The microbiological experiments were conducted in triplicate on each group.

### Determination of colony-forming units (CFUs)

The *S. oralis* culture ability on the different surfaces was determined by measuring the number of CFU of each sample. After washing with phosphate buffer solution (PBS) to remove unattached cells, the discs were inserted in a sterile test tubes containing 1 mL of PBS.

Each test tube was placed into the water of a 40 kHz ultrasonic cleaning bath (Euronda, Italy) for 4 min followed by vortex mixing for 2 min to remove the bacteria attached on the surface of each disc.

Microscopic observations, through Live/Dead staining, prior to plating confirmed that the microbial suspension consisted of a mixture of single, viable microbial cells (data not shown). Then, selected 10-fold dilutions were plated on TSA plates and incubated overnight al 37 °C, followed by counting of CFU/mL. According to the above results, the number of adhered viable bacteria on the surface of the specimen was calculated.

### Biofilm biomass assay

For the determination of the biofilm biomass (comprising bacterial cells/extracellular polymeric substances) after 24 and 48 h, the different types of discs were stained with crystal violet.

In particular, the samples were washed 3 times with PBS, fixed by air drying, stained with Crystal Violet 0.1% (Sigma Aldrich, Milan, Italy) for 1 min and, washed with PBS to remove excess stain. They were, then, dried for 2 h at 37 °C. The crystal violet was eluted with ethanol for reading. After 10 min the samples were removed and the biofilm formation was then quantified by measuring absorbance at 570 nm with a microplate reader (SAFAS, Munich, Germany).

The absorbance of the eluted stain is proportional to the concentration of biofilm biomass formed on the sample surface.

### Cell viability assay

For the evaluation of cells viability, the *S. oralis* cells and the developed biofilm on discs surfaces were examined with a BacLight Live/Dead Viability Kit (Molecular Probes, Invitrogen detection technologies, USA).

SYTO 9 stained viable cells with a green fluorescent signal, and propidium iodide (PI) stained the cells with damaged membrane in red.

Then, attached bacteria to each surface were washed with PBS and stained at room temperature for 15 min in the dark, as indicated by manufacturer. The images observed at fluorescent Leica 4000 DM microscopy (Leica Microsystems, Milan, Italy) equipped with a halogen lamp, Neoplan 100/1.25 oil objective and 1713 filter cube (fluorescein; 490/510/520 nm), were recorded at an emission wavelength of 500 nm for SYTO 9 (green), and at 635 nm for propidium iodide (red fluorescence). The enumeration was performed by three blinded microbiologists by using an image analysis software (LEICA QWin) though the examination of at least 10 random fields of view each, as indicated by several Authors [23, 24, 26, 27].

For this detection, 9 discs (3 for each sample surface) were analyzed in triplicate for a total of 27 discs.

### Statistical analysis

Statistical analysis was performed by using SPSS for Windows version 21 (IBM SPSS Inc., Chicago, IL, USA), analysis of variance (ANOVA) and the Least Significant Difference (LSD) test were used to compare the parameters analyzed in the study for intra- and inter-group analysis.

CFUs were expressed as log_10_CFU/mL. Pearson correlation was used to assess the relationship between the materials’ features and microbiological results.

Data were analyzed using linear regression and descriptive statistics. *p* values less than 0.05 were considered significant.

## Results

**Fig. 1:**
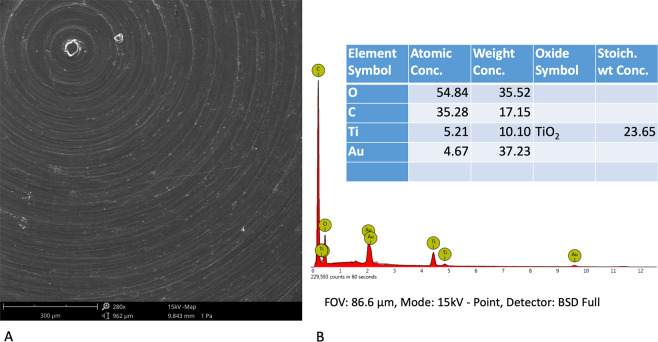
Superficial micro-topography of PEEK samples observed at SEM (**a**) with the relative EDS spectra (**b**)

**Fig. 2:**
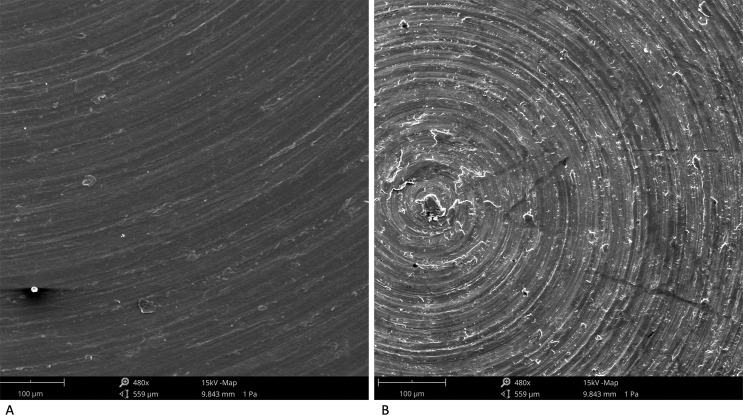
Superficial micro-topography of PEEK samples at higher magnification (**a**), compared with titanium machined (**b**)

**Fig. 3:**
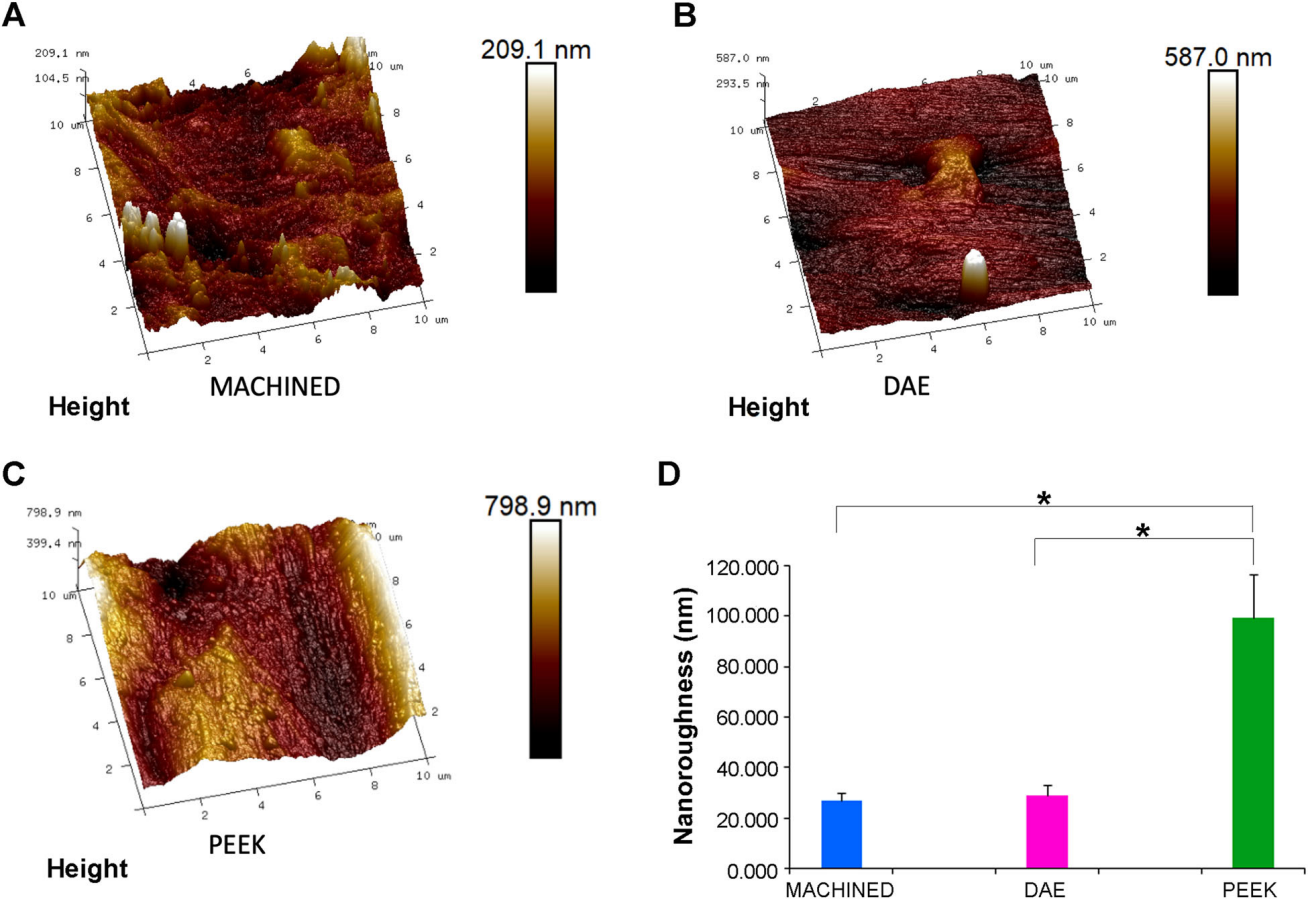
3D reconstruction of AFM images of machined Ti (**a**), DAE Ti (**b**) and PEEK (**c**). Average Ra (± standard deviation) calculated on an area comprised between 10 μm*10 μm. Statistically significant differences (*p < 0.05) between PEEK and the other two groups (**d**)

The EDS analysis showed that PEEK samples were mainly composed by Carbonium and Oxygen (Fig. 1a). The SEM observations confirmed that the microarchitecture of PEEK samples (Figs 1, 2a) were very similar to those of machined titanium (Fig. 2b). On the contrary, AFM images (Fig. 3a–c) and average nano-roughness, Ra (Fig. 3d) have shown that PEEK samples were characterized by a higher nano-heterogeneity and Ra in respect to the other samples, *p* = 0.010 (ANOVA).

LSD test has shown significant differences, *p* = 0.005, for Ra when comparing PEEK (99.550 ± 17.171 nm) *vs* machined (26.800 ± 3.211 nm) and PEEK *vs* DAE (28.833 ± 4.533 nm), *p* = 0.010. On the contrary, there were no significant differences among machined *vs* DAE, *p* = 0.929.

**Fig. 4:**
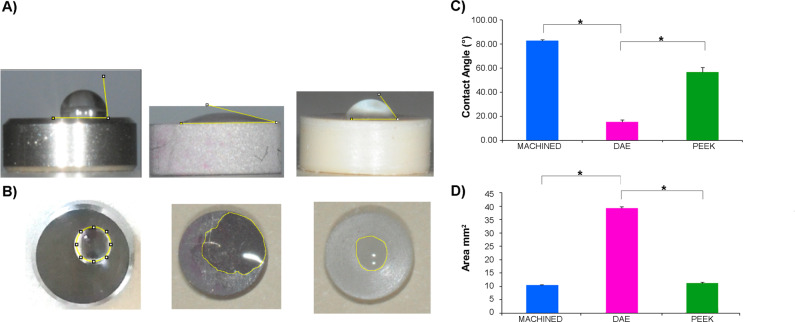
**a** Static contact angles with the sessile drop method of machined, DAE and PEEK. **c** Average water contact angle (*p-value < 0.05). **b** The wetted area of machined, DAE, PEEK. **d** Average wetted area of the samples (*p-value < 0.05)

The wetting analysis (Fig. 4) of the discs have shown that all samples could be considered as hydrophilic, because water contact angles were always less than 90°. ANOVA found significant differences (*p* < 0.001) at extra-group analysis for both water contact angle (WCA) and wetted area. DAE samples (15.666 ± 1.527°) were characterized by significant lower WCA, *p* < 0.001, in respect to PEEK (57.000 ± 3.605°) and to machined (83.001 ± 1.000°), Fig. 4c. The LSD analysis found statistically significant differences among all the groups, *p* < 0.001.

The wetted area was significantly higher on DAE discs (40.724 ± 0.455 mm^2^), in respect to PEEK and machined samples (*p* < 0.001), Fig. 4d. On the contrary, there were no significant differences among the wetted area measured in machined (10.994 ± 0.186 mm^2^) *vs* PEEK (11.648 ± 0.457 mm^2^).

**Fig. 5:**
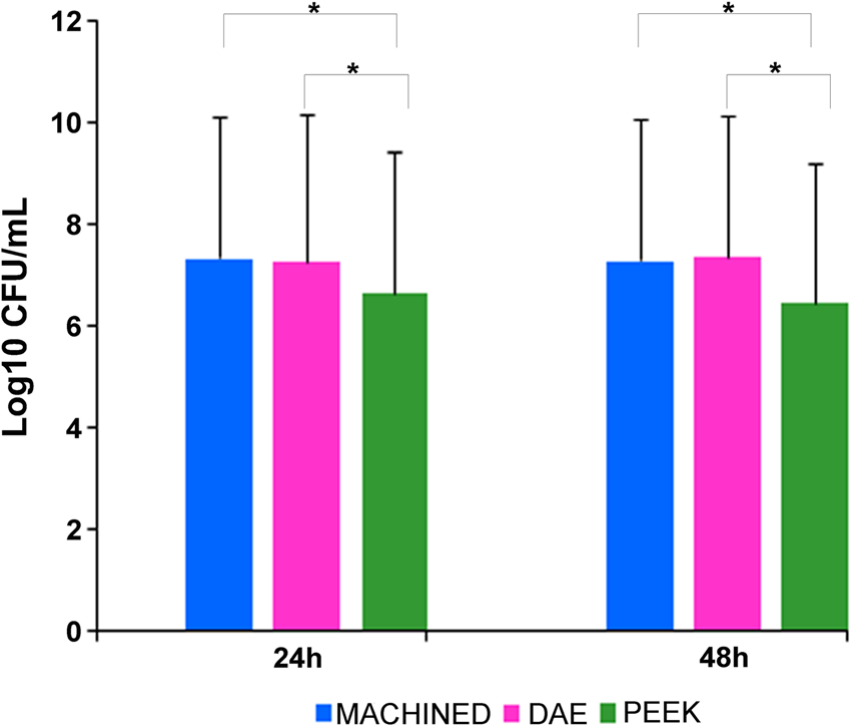
Comparison of the viable adherent bacteria Log_10_ CFU/mL of adherent *S. oralis* on titanium discs machined, DAE and PEEK ones, after 24 and 48 h of incubation (error bars = ±standard deviation)

As shown in Fig. 5, CFUs analysis displayed that PEEK was characterized by significant lower viable *S. oralis* counts when compared to machined and DAE samples, both at 24 and 48 h. In particular, the levels at 24 h of CFUs/mL expressed in log_10_ were 7.337 ± 3.320, 7.294 ± 3.290 and 6.655 ± 3.330 for machined, DAE, and PEEK, respectively. ANOVA has shown significant differences at extra-group analysis (*p* < 0.001) at 24 and 48 h. In particular, LSD test has shown at 24 h significant *p*-value when comparing PEEK *vs* machined (*p* < 0.001) and PEEK *vs* DAE (*p* = 0.002).

On the contrary, there were no significant differences among machined and DAE discs regard the number of viable *S. oralis* (*p* = 0.590). A similar trend was maintained at 48 h: PEEK (6.456 ± 2.721 log_10_CFU/mL) was characterized by significant lower values (*p* < 0.001) in respect to machined (7.308 ± 2.801 log_10_CFU/mL) and DAE (7.374 ± 2.911 log_10_CFU/mL). No significant differences were found between machined and DAE (*p* = 0.447) for CFU at 48 h. In particular, CFUs reduction on PEEK samples was 80% at 24 h and 85% at 48 h in respect to the machined ones. Moreover, PEEK samples were characterized by a reduction percentage of CFUs in respect to the DAE of 77 and 88%, at 24 and 48 h, respectively.

**Fig. 6:**
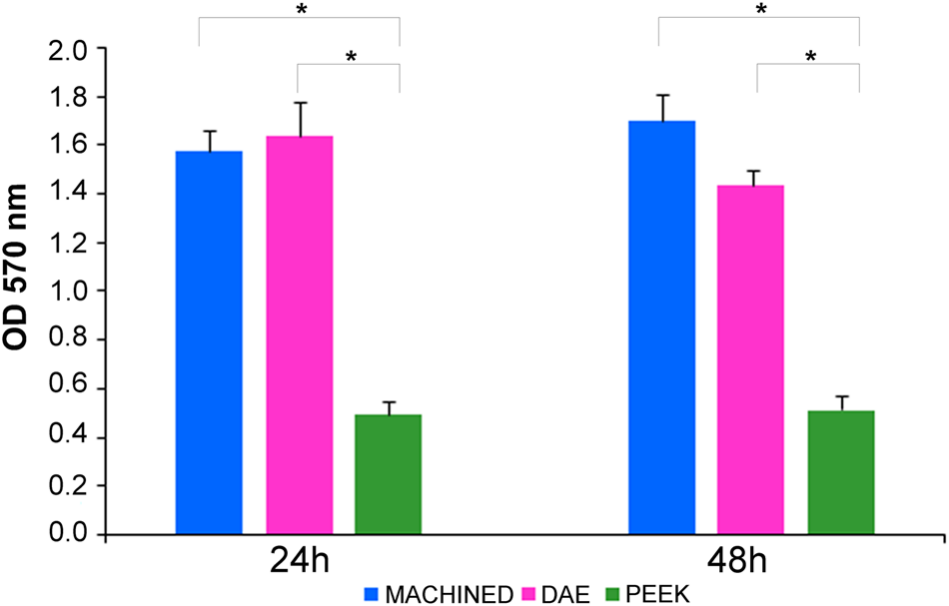
Comparison of total biofilm biomass for the studied *S. oralis* biofilm developed on titanium discs machined, DAE and PEEK ones, after 24 and 48 h of incubation (error bars = ± standard deviation),determined by crystal violet staining (*p-value < 0.05)

The same trend was shown for the biofilm biomass analysis (Fig. 6). ANOVA has shown significant differences at extra-group analysis (*p* = 0.002) at 24 and 48 h (*p* < 0.001). LSD test has shown at 24 h significant *p*-value (*p* < 0.001) for biomass when comparing PEEK (0.499 ± 0.052) *vs* machined (1.580 ± 0.086), and PEEK *vs* DAE (1.638 ± 0.143). On the contrary, there were no significant differences among machined *vs* DAE discs regard the biomass (*p* = 0.830). A similar trend was maintained at 48 h: LSD test has shown significant *p*-value (*p* < 0.001) when comparing PEEK (0.516 ± 0.059) *vs* machined (1.700 ± 0.113), and PEEK *vs* DAE (1.438 ± 0.062). On the contrary, there were no significant differences among machined *vs* DAE discs regard the biomass (*p* = 0.298).

**Fig. 7:**
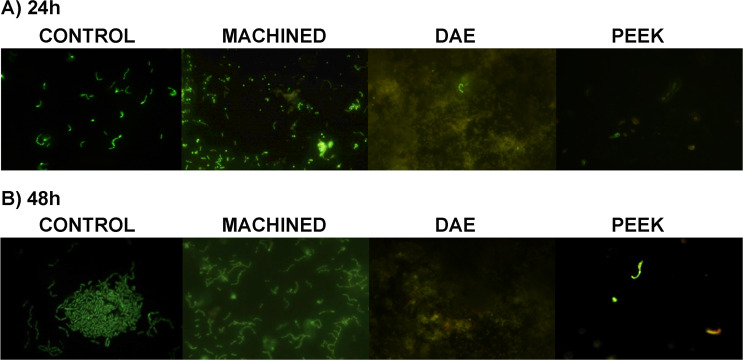
Representative Live/Dead images of *S. oralis* adhesion on titanium discs machined, DAE and PEEK ones after 24 and 48 h of incubation (live cells appeared green, death cells were red) (*p-value < 0.05). All formed biofilms on different material surfaces were compared to the controls group that were the untreated samples obtained with the identical methodologies in every way except for the use of one of tested surfaces but on polystyrene surface. Original magnification, 1000 X

Live/Dead images of biofilm obtained using fluorescent microscopy showed that the total cell biomass increased over time (Fig. 7). PEEK discs demonstrated fewer cells and more vacant areas, both 24 and 48 h.

**Fig. 8:**
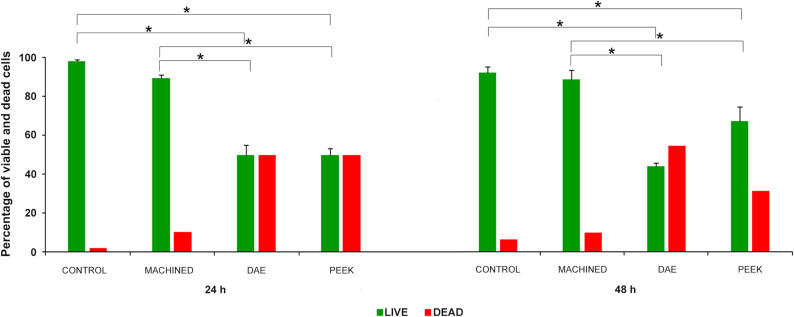
Percentage of viable (green) and dead (red) cells in the formed biofilm on machined, DAE and PEEK with Live/Dead staining. All formed biofilms on different material surfaces were compared to the controls group that were the untreated samples obtained with the identical methodologies in every way except for the use of one of tested surfaces but on polystyrene surface. The control group was used to evaluate the effect of the each treatment(*p-value < 0.05)

The observations showed a more organized biofilm at 48 h in all groups. However, the percentage of viable cells (green cells) in DAE and PEEK group was significantly lower in respect to machined discs and controls. Considerable percentage of dead cells (red cells) occurred on DAE and PEEK discs (Fig. 8). ANOVA found significant differences at extra-group analysis at 24 and 48 h.

The calculated percentage values of viable cells at 24 h were 98 ± 1.0, 90 ± 1.5, 50 ± 5.0 and 50 ± 3.5 for control, machined, DAE and PEEK, respectively.

LSD analysis found *p* < 0.001 when comparing all groups, except for machined *vs* controls (*p* = 0.031). On the contrary, there were no differences between DAE and PEEK (*p* = 1.000). The calculated percentage values of viable cells at 48 h were 94 ± 3.2, 90 ± 5.0, 45 ± 1.5, and 68 ± 7.6 for control, machined, DAE and PEEK, respectively.

LSD analysis found *p* < 0.001 when comparing all groups, except for machined *vs* controls that were no significant (*p* = 0.312).

The inter-group analysis showed a significant increase of viable cell from 24 to 48 h (*p* < 0.001) in PEEK samples.

The results of the Pearson analysis are showed on Table 1; a significant inverse relationship was shown between the values of the water contact angle and the measured CFUs at 24 h (r = −0.866, *p* < 0.01). A significant inverse relationship was shown between biomass at 24 h and the values of the wetted area (r = −0.723, *p* < 0.05) and Ra (r = −0.671, *p* < 0.05).

**Table 1:** The values of the Pearson’s correlation of the parameters analyzed in the study

			WCA	WETTED AREA	Ra
24 h	CFUs/mL	Pearson’s correlation (r)	−0.866	−0.572	−0.551
		Sig. (2-tails)	0.003**	0.107	0.079
	BIOMASS	Pearson’s correlation (r)	0.145	−0.723	−0.671
		Sig. (2-tails)	0.709	0.028*	0.024*
48 h	CFUs/mL	Pearson’s correlation (r)	0.041	−0.910	−0.820
		Sig. (2-tails)	0.917	0.001**	0.002**
	BIOMASS	Pearson’s correlation (r)	−0.200	−0.875	−0.746
		Sig. (2-tails)	0.606	0.002**	0.008**

*The correlation is significant at 0.05 (2-tails). **The correlation is significant at 0.01 (2-tails)

The values of CFUs and biomass at 48 h were significantly correlated, *p* < 0.01, with an inverse relationship with the wetted area and Ra.

## Discussion

The aim of this study was to evaluate the biofilm formation of a common bacterial pioneer on PEEK samples and to compare results with two titanium IV grade surfaces, machined and DAE, commonly used in implantology.

In order to understand the role of the different features of the samples on biofilm formation, a material characterization at nano- micro- and chemical level, was performed in all specimens.

The SEM observations confirmed that the microarchitecture of PEEK samples were very similar to those of machined titanium, but the EDS analysis confirmed the typical spectra of PEEK samples that were mainly composed by Carbonium and Oxygen, as previously shown by Ma et al. [28].

The value of Ra measured by the AFM analysis showed that PEEK discs were characterized by a significant higher nano-roughness in respect to machined Ti, although the discs were produced both by turning and were subjected to the same decontamination phase.

The measurements on Ti samples were not in accordance with a previous study of Bathomarco et al. [29], but it is important to highlight that, in the present study, the values of Ra were calculated as a mean value of random portions of the sample of 10*10 µm, contrarily to those authors that analyzed a scanning sizes of 100*100 µm.

The microbiological analysis showed that *S. oralis* biofilm formation was significantly lower on PEEK discs in respect to the other two groups, as resulted by CFUs count and biofilm biomass evaluation, demonstrating a higher anti-bacterial and anti-adhesive effect.

The Live/Dead images showed that the ratio between viable cells/dead ones on PEEK samples was significantly lower when compared to control and Ti machined samples, both at 24 and 48 h, confirming the bactericidal effect of the material.

These results are more significant considering that Ti machined and PEEK discs were characterized by similar wetting properties and hydrophilicity.

There are only few studies in literature that analyzed biofilm formation on PEEK surfaces. Barkarmo et al., have recently shown results about biofilm formation of different reference strains on PEEK and titanium surfaces at 72 and 120 h [30]. They concluded that no significant differences were found between smooth PEEK, machined commercially pure Ti, and Ti_6_Al_4_V and blasted PEEK was characterized by a higher biofilm formation. As highlighted by Barkarmo et al., the influence of superficial topography of the sample after 72 and 120 h is minimal and the significant differences that they found between smooth and blasted PEEK is more probably influenced by other factors, like the different chemical composition of the surfaces [30].

The experiments conducted in the present paper underlined the specific anti-adhesive action of PEEK surfaces, pre-treated by saliva mimicking the oral cavity environment against an early colonizer such as on a clinical isolate of *S. oralis*.

The present results are very encouraging, because the antibiofilm effect exerted by PEEK samples in the first 24 and 48 h represent a guarantee of protection against bacteria both immediately after implant insertion, but also later, during the implant function, because patients are commonly instructed to perform, as for natural teeth, oral hygiene manoeuvres, at least twice a day [31, 32].

To best our knowledge this is the first work that has shown the antibiofilm effect of PEEK at 24 and 48 h, after pre-incubating disc with saliva, in order to form an acquired pellicle.

There were no significant differences for biofilm formation between machined and DAE titanium surfaces: this is the further confirmation, that as shown by Daubert et al. the superficial roughness influences the bacterial adhesion and it is fundamental only in the first hours [33].

Finally, the obtained results are more in accordance with Hahnel et al. that compared the multispecies biofilm formations on discs of different materials previously incubated with saliva [27]. The biomass of PEEK samples evaluated at 20 and 44 h were significantly lower respect other materials, among machined titanium.

In addition, in the present study, it is confirmed the great importance of both wettability of the material and nano-roughness of the samples in influencing both CFUs and biomass at 24 and 48 h. The impact of these results on the clinical practice is particularly remarkable. Indeed, it has been shown that biofilm formation is the first step for the etiology of implant disease and also the current therapies used to treat peri-implantitis, consist mainly in biofilm removal, implant decontamination, and eventually surgical intervention that could permit the elimination of intrabony defects where plaque could accumulate [34–36]. These results are important also for maxilla-facial surgery and ortopaedics, because PEEK can be used as a substitute material for bone tissue engineering and currently about 20% of surgery failures are caused by the infections [37].

## Conclusions

In the complex, both wettability and nano-roughness of PEEK are able to significantly affect the CFUs and biofilm biomass.

PEEK discs showed a remarkable decrease in the *S. oralis* biofilm formation, demonstrating bactericidal and/or anti-adhesive effect, respect machined and DAE titanium.

The anti-adhesive and antibacterial properties showed by PEEK at 24 and 48 h against a pioneer such as *S. oralis*, could have an important role in the prevention of all pathologies connected with biofilm formation, like peri-implantitis in dentistry or prosthetic failures in orthopedics.
